# Vitamin D Is a Major Determinant of Bone Mineral Density at School Age

**DOI:** 10.1371/journal.pone.0040090

**Published:** 2012-07-02

**Authors:** Minna Pekkinen, Heli Viljakainen, Elisa Saarnio, Christel Lamberg-Allardt, Outi Mäkitie

**Affiliations:** 1 Folkhälsan Institute of Genetics, University of Helsinki, Helsinki, Finland; 2 Calcium Research Unit, Department of Food and Environmental Sciences, University of Helsinki, Helsinki, Finland; 3 Department of Pediatrics, Children’s Hospital, Helsinki University Central Hospital and University of Helsinki, Helsinki, Finland; Faculté de Médecine de Nantes, France

## Abstract

**Background:**

Vitamin D insufficiency in children may have long-term skeletal consequences as vitamin D affects calcium absorption, bone mineralization and bone mass attainment.

**Methodology/Principal Findings:**

This school-based study investigated vitamin D status and its association with vitamin D intake and bone health in 195 Finnish children and adolescents (age range 7–19 years). Clinical characteristics, physical activity and dietary vitamin D intake were evaluated. Blood and urine samples were collected for serum 25-hydroxyvitamin D (25-OHD) and other parameters of calcium homeostasis. Bone mineral density (BMD) and body composition were measured with dual-energy X-ray absorptiometry (DXA). Altogether 71% of the subjects were vitamin D insufficient (25-OHD <50 nmol/L). The median 25-OHD was 41 nmol/L for girls and 45 nmol/L for boys, and the respective median vitamin D intakes 9.1 µg/day and 10 µg/day. In regression analysis, after adjusting for relevant factors, 25-OHD concentration explained 5.6% of the variance in lumbar BMD; 25-OHD and exercise together explained 7.6% of the variance in total hip BMD and 17% of the variance in whole body BMD. S-25-OHD was an independent determinant of lumbar spine and whole body BMD and in magnitude surpassed the effects of physical activity.

**Conclusions/Significance:**

Vitamin D insufficiency was common even when vitamin D intake exceeded the recommended daily intake. Vitamin D status was a key determinant of BMD. The findings suggest urgent need to increase vitamin D intake to optimize bone health in children.

## Introduction

The majority of bone mass is attained during adolescence and young adulthood, with almost 90% of the skeletal mass accumulated by age 18 [1]. Many factors influence bone mass accrual during growth, including genetic factors, gender, race, endocrine and mechanical factors, some pharmacological agents and dietary factors, especially calcium and vitamin D intake [2].

Vitamin D is important for calcium metabolism and accretion of bone mass during growth [3]–[6]. Serum concentration of the hydroxylated form, 25-hydroxyvitamin D (25-OHD), indicates vitamin D status. 25-OHD is converted to 1,25-dihydroxy vitamin D in the kidney but also in an autorine/paracrine mode in many other cell types [7]. Some epidemiological studies have demonstrated the importance of vitamin D for bone health, immune functions and neuronal system [8] and for prevention of cardiovascular diseases [9] and certain cancers [10]. Vitamin D deficiency has also been associated with autoimmune diseases such as type I diabetes [11] and with multiple sclerosis [12].

Epidemiological studies over the last decade have shown that vitamin D deficiency is common in children and especially adolescents. In these age groups, complications of vitamin D deficiency are likely to appear with significant delay and deficiency may thus remain unnoticed [13]. For children and adolescents, serum 25-OHD concentration of 37.5 nmol (15 ng/ml) was previously considered as the lower limit for vitamin D sufficiency. More recently, it has been widely agreed that a serum concentration greater than 50 nmol/L (>20 ng/ml) is physiologically more appropriate [13]–[15] and associates with many health benefits. Significant seasonal variation and inadequate vitamin D status especially during winter months has been demonstrated in northern latitudes [4], [5], [16]. Meta-analysis suggested 25-OHD concentrations to be higher in subjects aged >15 years than in younger children [17].

Physical activity during childhood and adolescence enhances bone mass accrual; the maximal effect may be achieved when activity begins at pre-puberty and continues through pubertal years [18]. Exercise also increases bone mass indirectly by increasing muscle mass and hence tension generated on bones [19]. Previous studies suggest that intensity rather than duration of the activity is important for bone mineral density (BMD) [20]. Most of the previous studies have assessed the skeletal effects of exercise and vitamin D separately and in small cohorts with a limited age range. In the present study we explored the impact of both vitamin D status and physical activity on skeletal health in a relatively large cohort of Finnish children and adolescents ranging in age from 7 to 19 years. Further, we investigated the association of vitamin D status with vitamin D intake. The findings suggest that the recommended vitamin D intake in this age group does not ensure vitamin D sufficiency.

## Results

Of the 195 subjects who participated in the present study, 62% (N = 120) were girls and 38% (N = 75) were boys. Cohort medians for age, pubertal stage, height, weight, total intake of calcium and vitamin D (combines intakes from diet and supplements) and physical activity are presented in Table 1. The median height-adjusted weight was in agreement with reference values; 18.3% were overweight (height-adjusted weight >20% - 40%) and 2.3% were obese (height-adjusted weight >40%). The median total intakes of calcium and vitamin D were in line with recommendations [21] in both genders. However, there was great variation in individual intakes, especially for vitamin D, and 34% of the subjects had vitamin D intake less than recommended (<7.5 µg/d). The participants were physically active: 70% of the study population had a physical activity score >17 and 28% had a score >20, which indicate more than 1.5 and 2 hours, respectively, of physical activity daily.

**Table 1:** Clinical characteristics and biochemical and bone densitometry findings in the 195 children and adolescents. P-values refer to the difference between girls and boys.

	Girls (n = 120)	Boys (n = 75)	P-values
Age	13.3 (7–19)	12.6 (8–18)	0.898
Pubertal stage			
- prepubertal	30	40	
- pubertal	36	12	
- postpubertal	54	23	
Height (Z-score)	+0.3 (−2.3–+2.8)	+0.5 (−1.8–+3.2)	0.573
Height-adjustedweight (%)	+3 (−25–+48)	+3 (−16–+92)	0.478
Calcium intake(mg/day)	1407 (477–3618)	1506 (743–2976)	0.528
Vitamin D intake(µg/d)	9.1 (2–27)	10 (3–24)	0.135
Physical activity score	18 (6.5–27)	19 (10–26)	0.605
S-25-OHD(nmol/L)	41 (18–82)	45 (17–77)	0.626
P-PTH (ng/L)^a^	39 (14–135)	38 (3–136)	0.890
P-Ca (mmol/L)^b^	2.33 (2.06–2.58)	2.32 (2.16–2.61)	0.115
P-Pi (mmol/L)^c^	1.37 (1.00–1.82)	1.47 (0.83–2.11)	0.678
P-ALP (Z-score)^d^	−0.9 (−3.3–+3.3)	0.0 (−4.2–+2.8)	0.087
U-Ca/U-Crea^e^(mmol/L/mmol/L)	0.15 (0.03–0.74)	0.16 (0.04–0.88)	0.077
LS Z-score	−0.1 (−2.2–+2.7)	0.2 (−1.3–+2.6)	0.928
Total hip Z-score	0.2 (−1.9–2.5)	0.1 (−1.6–1.9)	0.806
WB Z-score	0.0 (−2.1–+2.4)	0.0 (−1.5–+2.4)	0.665
Lean body mass(Z-score)	−0.13 (−1.9–+1.9)	−0.31 (−2.3–+1.7)	0.464
Fat % (Z-score)	−0.5 (−2.8–3.5)	−0.3 (−2.4–3.9)	0.185
BMI (Z-score)	−0.1 (−2.2–2.5)	0.3 (−1.9–3.5)	0.392

All values are median; range in parentheses. LS =  lumbar spine, WB =  whole body, BMI =  body mass index.

a Reference ranges, PTH, 8–73.

b Reference ranges, P-Ca, 2.05–2.7 (6–10 age), 2.15–2.7 (11–16 age), 2.25–2.65 (17 age),

2.15–2.51 (since 18 age).

c Reference ranges, P-Pi, 1.2–1.8 (2–12 age), 1.1–1.8 (13–16 age), 0.8–1.4 (17 age), 0.71–1.53 (male, 18–49 age), 10.76–1.41 (female, since 18 age).

d Reference ranges, P-ALP (Z-score), −2.0–+2.0^e^ Reference ranges, U-Ca/U-Crea, <0.70.

**Figure 1:**
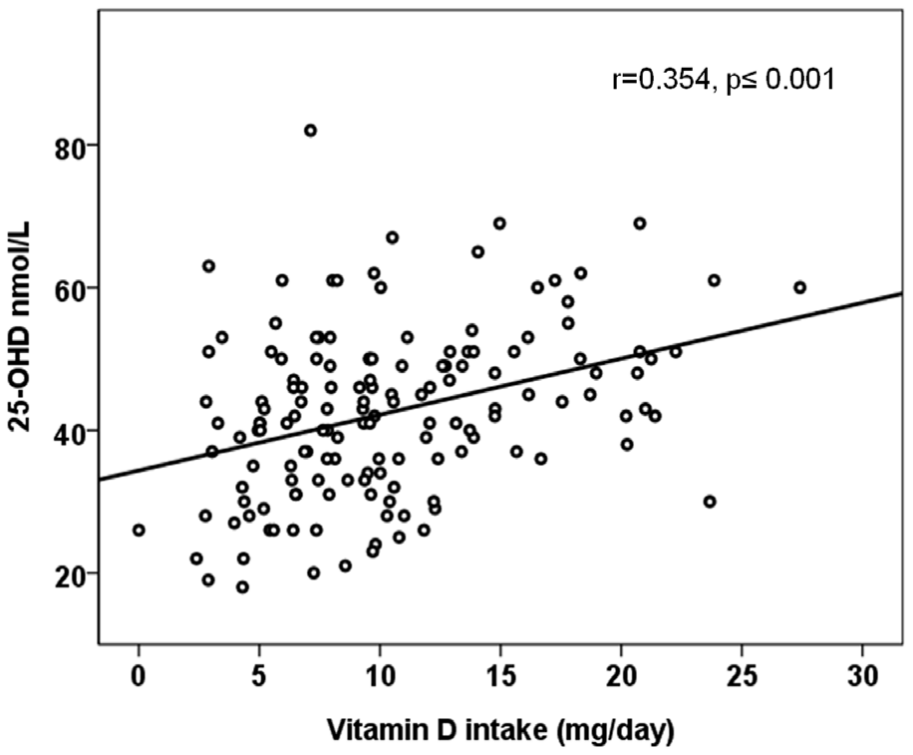
Association between vitamin D intake and serum 25-OHD concentration.

**Figure 2:**
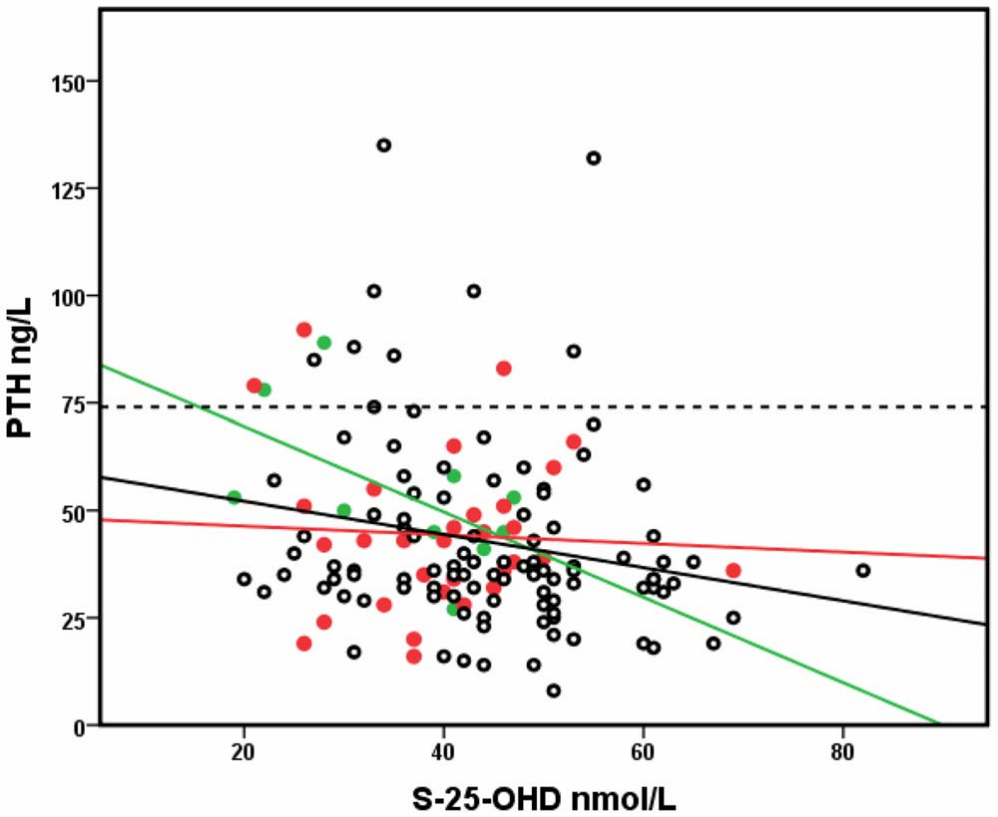
Association of serum 25-OHD, PTH concentration, and calcium intake. Calcium intake in tertiles is marked with colors: <800 mg/day (green circles), 800–1200 mg/day (red circles) and >1200 mg/day (black circles), Pearson correlations between S-25-OHD and PTH r = −0.563, p = 0.09; r = −0.054, p = 0.772, r = −0.248, p = 0.017 respectively. The PTH reference line is marked with the broken line.

The median serum 25-OHD, plasma parathyroid hormone (PTH), Ca, Pi, alkaline phosphatase (ALP) and urine Ca/Crea are presented in Table 1. We observed that 71% of the study population had serum 25-OHD concentration below 50 nmol/L (73.5% of the girls, 68.5% of the boys), the median being 41 nmol/L for girls and 45 nmol/L for boys (range 17–82 nmol/L). The median vitamin D intakes were 9.1 and 10 µg/day in girls and boys, respectively. There was a positive correlation between the total intake of vitamin D and serum 25-OHD (r = 0.217, p = 0.003), controlling for the date of sampling, and an inverse association between serum 25-OHD and plasma PTH (r =  −0.196, p = 0.02) after controlling for calcium intake. The assosiation between serum 25-OHD with total vitamin D intake is shown in Figure 1. There was a significant difference in PTH levels according to vitamin D status, the concentration being highest when serum 25-OHD was ≤37.5 nmol/L (PTH 51.7 ng/L), intermediate when serum 25-OHD was between 37.5–50 nmol/L (41.3 ng/L) and lowest when serum 25-OHD was >50 nmol/L (39 ng/L) (ANOVA; p = 0.001). Vitamin D intake also differed according to vitamin D status (ANOVA; p<0.001). No correlation was observed between plasma ALP and either serum 25-OHD or PTH concentrations. Similarly, serum 25-OHD did not correlate with urine Ca/Crea ratio. There was a trend in serum 25-OHD according to date of sampling, the concentrations being higher in late autumn than in early spring (r = −0.131, p = 0.074). The lowest PTH concentrations were observed in subjects with calcium intake >1200 mg/day, with only a small effect by 25-OHD status, whereas the PTH values tended to be higher in those with 25-OHD values <25 nmol/L and calcium intake <800 mg/day (Figure 2). Calcium intake did not affect the association between serum 25-OHD and PTH (ANCOVA p = 0.046, using calcium intake groups as covariant).

**Figure 3:**
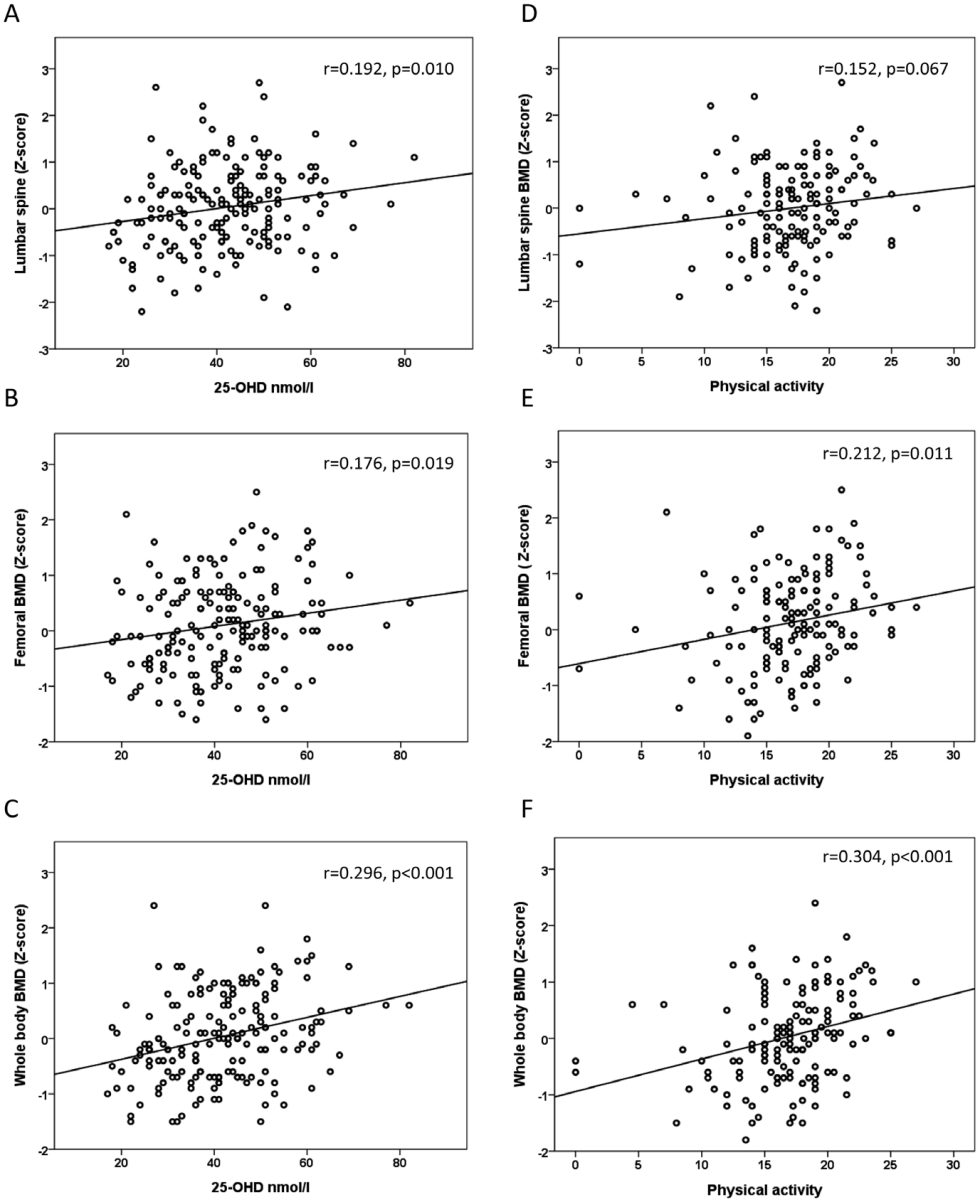
Association between vitamin D status and physical activity on bone mineral density (BMD). Correlations between serum 25-OHD concentrations and lumbar spine (**A**), total hip (**B**), and whole body (**C**) bone mineral density (BMD) Z-scores, adjusted for age and gender. Correlations between physical activity and lumbar spine (**D**), total hip (**E**), and whole body (**F**) BMD Z-scores, adjusted for age and gender. Physical activity scores of 13.5, 17 and 20 correspond to 1, 1.5 and 2 h daily activity.

No difference between the genders was observed in the median lumbar spine (LS), total hip, and whole body (WB) BMD, lean body mass and fat % Z-score (Table 1). The LS, total hip and WB BMD Z-scores differed according to vitamin D status (r = 0.192, p = 0.010, r = 0.176, p = 0.019, r = 0.296, p<0.001, respectively) (Figure 3). Physical activity also had an effect on BMD, the total hip and WB BMD Z-score differed according to physical activity (r = 0.212, p = 0.011, r = 0.304, p<0.01, respectively) but no difference was noticed in LS BMD Z-score (r = 0.152, p = 0.145) (Figure 3).

In order to determine the factors that affect LS, total hip and WB BMD Z-scores in children and adolescents, a stepwise regression analysis was performed. In this regression model, pubertal stage, height Z-score, fat % Z-score, lean body mass, calcium intake, serum 25-OHD and PTH concentrations and physical activity scores were included. The regression model accounted for 28.6% of the variance (adjusted R^2^) in LS BMD Z-score, 18.6% of the variance in total hip BMD Z-score and 22.4% of the variance in WB BMD Z-score (Table 2). Among all the independent variables, fat % Z-score, serum 25-OHD concentration, height Z-score, lean body mass and exercise were the significant determinants of LS BMD Z-score. Similarly, height Z-score, serum 25-OHD concentration, fat % Z-score and exercise were the significant determinants of total hip BMD, while height Z-score, serum 25-OHD concentration and exercise determined WB BMD Z-score.

**Table 2:** Linear stepwise regression analysis for determinants of lumbar spine, total hip and whole body BMD Z-score.

	Lumbar spine BMD (Z-score)			Total hip BMD (Z-score)			Whole body (Z-score)		
	*Adjusted r^2^*	*ß*	*P*	*Adjusted r^2^*	*ß*	*P*	*Adjusted r^2^*	*ß*	*P*
Regression model	**0.286**		**<0.001**	**0.186**		<**0.001**	**0.224**		**<0.001**
Serum 25-OHD (nmol/L)	**0.056**	0.198	**0.009**	**0.019**	0.157	**0.049**	**0.099**	0.285	**<0.001**
Height (z-score)	**0.065**	0.186	**0.017**	**0.086**	0.241	**0.003**	**0.054**	0.243	**0.002**
Calcium intake (mg/day)	–	0.034	0.667	-	0.062	0.461	-	0.071	0.382
Fat % (z-score)	**0.107**	0.058	**<0.001**	**0.024**	0.185	**0.023**	-	−0.106	0.176
Lean body mass (kg)	**0.032**	0.253	**0.002**	-	−0.037	0.661	-	−0.102	0.217
ft-PTH (ng/L)	–	−0.088	0.250	-	−0.047	0.563	-	−0.075	0.337
Pubertal stage	–	0.025	0.817	-	−0.071	0.397	-	−0.092	0.257
Exercise (physical activity score)	**0.029**	0.197	**0.013**	**0.057**	0.261	**0.001**	**0.071**	0.254	**0.001**

ß values are standardized. Determinants with bold p-value are included in the model.

## Discussion

Adequate vitamin D and calcium intake are important for bone mass accrual and long-term skeletal health. This cross-sectional study indicates that among Finnish school children and adolescents vitamin D insufficiency is alarmingly prevalent, only 29% of the 195 evaluated subjects having sufficient (>50 nmol/L) vitamin D status during the school year. Furthermore, serum 25-OHD was an independent determinant of lumbar spine and whole body BMD and in magnitude surpassed the effects of physical activity.

The average total vitamin D intake was 10.4 µg/day and thus in accordance with the present Nordic recommendation (7.5 µg/day) [21]. However, in 34% of the subjects the intake was below 7.5 µg/day. Vitamin D intake correlated strongly with serum 25-OHD concentration. Vitamin D intake was on average 12.3 µg/day in vitamin D sufficient subjects and 8.5 g/day in subjects with S-25-OHD below 37.5 nmol/L. On the other hand, despite significantly higher total intakes (up to 27 µg/day) in some subjects, only one individual of the total cohort (0.5%) had S-25-OHD above 80 nmol/L, which based on some adult studies may be optimal for overall benefits of vitamin D [22]. These findings indicate that the recommendation for vitamin D intake should be significantly higher than the current recommendation.

The key finding of the present study is that insufficient serum 25-OHD concentrations associate with lower BMD Z-scores and its effect was greater than that of physical activity at LS and WB. S-25-OHD explained up to 9.9% of the variation in BMD Z-score. Low S-25-OHD impairs calcium absorption and the unoptimal calcium balance triggers the release of PTH. Continuously elevated PTH in turn induces mobilization of calcium from bone, resulting in a reduction of bone mass and consequently in increased risk of fractures [23]. The observed suboptimal vitamin D levels may thus have life-long skeletal consequences.

In 70% of our subjects daily physical activity exceed 1.5 hour per day which meets the recommendation for this age-group [20]. Physical activity determined 5.7% and 7.1% of the variability in total hip and WB BMD Z-score. Especially in weight bearing sites the effect of physical activity is pronounced [24]. This finding is in line with many studies, which have demonstrated that the growing skeleton responds to everyday physical activity by increased bone mineral accrual and density [1], [25]. There was a positive trend between vitamin D status and physical activity, the subjects with higher physical activity also having higher S-25-OHD values. Similar association has been observed in some other studies [26]. Rather than causality, this may reflect the overall lifestyle.

Calcium intake was relatively high (medians for girls and boys 1407 and 1506 mg/day, respectively), reflecting the high consumption of milk products in Finland [27]. In spite of the high mean intake, there was considerable variation in calcium intake, allowing us to examine the impact of calcium intake in tertiles (<800, 800–1200, and >1200 mg/day). There was an inverse correlation between vitamin D status and PTH concentration regardless of calcium intake. Highest PTH values were observed in subjects with lowest 25-OHD. Our results are supported by Steingrimsdottir et al. [28].

Although fat% and height Z-score appeared to be the strongest determinants of LS and total hip BMD Z-score, 25-OHD status explained 5.6% and 1.9% of the variations, respectively. Based on our findings, S-25-OHD is the most important determinant of WB BMD Z-score. In a previous study on 178 Finnish girls aged 14–16 years, ulnar and radial BMDs adjusted for exercise were slightly lower in those with S-25-OHD ≤40 nmol/L than in those above this cut-off level [25]. In another study with 171 Finnish girls aged 9–15 years, the adjusted 3-year accumulation in LS BMD was 4% smaller in those with serum 25-OHD levels below 20 nmol/L than in those with 25-OHD exceeding 37.5 nmol/L; the adjusted change in LS BMD was 27% greater in girls in the highest vitamin D tertile [3]. In 220 Finnish young men aged 18.3–20.6 years a positive correlation was found between S-25-OHD and LS, femoral neck, trochanter and total hip bone mineral content after adjusting for age, height, weight, exercise, smoking, calcium, and alcohol use [29].

The strenght of this study was that we were able to evaluate concomitant effects of sufficient serum 25-OHD concentration and physical activity on bone health. Furthermore, the cohort was relatively large and included children aged from 7 to 19 years. Our study has also some limitations. This cross-sectional study was geographically limited to Helsinki area in southern Finland. Therefore the findings can not directly be generalized to other regions but may well be representative of other Nordic and North European countries. The study included more girls than boys and the distribution of pubertal stage varied between the genders. The International Society for Clinical Densitometry recommends that in children total body less head BMD Z-scores rather than total body BMD Z-scores are used [30]. However, we used only total body values since no normative data were available to calculate total body less head Z-score values. It is unlikely that this has a significant effect on the findings. Previously serum 25-OHD concentrations were measured with HPLC, which could give up to 5% higher results compared with All-Laboratory Trimmed Mean (ALTM) [31]. This would however strengthen our finding of high prevalence of vitamin D deficiency. Our analysis included several factors known to influence bone health but due to the cross-sectional nature we were not able to analyze how these and other factors, including growth and development and overall health, impact bone mass accrual over time. Furthermore, while genetic factors have a major effect on skeletal health our cohort was not large enough to explore these aspects.

In conclusion, this school-based study showed that vitamin D insufficiency is alarmingly common in children and adolescents, even in subjects whose vitamin D intake meets the recommended daily intake. Vitamin D status was an important determinant of BMD, its effect being greater than that of physical activity in our study. In order to guarantee optimal peak bone mass attainment significant efforts should be placed on ensuring adequate vitamin D status in all children by revising the national recommendations for vitamin D intake. Further studies are needed to define the optimal vitamin D dose and the potential beneficial skeletal effects and overall health benefits of vitamin D sufficiency in growing children.

## Materials and Methods

### Ethics Statement

Ethics Committee approval for this study was obtained at the Helsinki University Hospital, Helsinki. The participants and their parents gave an informed written consent before entering the study.

### Study Population

A total of 195 children and adolescents - 120 girls (median age 13.3, range 7.4–18.8) and 75 boys (median age 12.6, range 7.7–18.1) - were included in this school-based cross-sectional study in the capital region of Helsinki (60°N), in southern Finland. The subjects were recruited from one primary and one secondary school in order to cover all age groups. Participation to this study, which assessed vitamin D levels and bone health, was offered to randomly selected school classes, aiming at greater than 60% participation rate in each participating class. Invitation letters, giving details about the study were given by the teachers to the students and their parents. All those willing to participate were included. More than 90% of the subjects were Caucasian.

### Clinical Data

The subjects completed a questionnaire on medical history, medications, overall health, age at menarche, use of vitamin D and calcium supplements and details about their physical activity. The questionnaire was given to the subjects during the first study visit, at the time of blood sampling and was returned to the researchers within two weeks. The questionnaire included a semi-quantitative food frequency questionnaire (FFQ), which has been validated for S-25-OHD and 3-day food records [32]–[34] and covers over 70 foods, to evaluate dietary intakes of vitamin D and calcium during the preceding month. The nutrient contents of the foods were calculated using the Finnish National Food Composition Database (Fineli®, version 2001, National Institute for Health and Welfare). Data on physical activity during two preceding years included regular every-day activities (e.g. walking to school), activity at school, and both guided and unguided leisure-time activities. Duration, frequency and intensity of activity sessions were recorded; both weight-bearing and non-weight bearing physical activity was included. A total physical activity score was obtained as a sum for a whole week. Physical activity scores of 13.5, 17 and 20 correspond to 1, 1.5 and 2 h daily activity, respectively, which meet the recommendation for this age group [21]. In addition, duration of each activity was multiplied with a weighting factor provided by Neville et al. [35] to obtain scores for intensity of physical activity and strenuous exercise, expressed as a total for a whole week. All forms were checked by the researchers and, if needed, information was clarified by interview. Height and weight were measured and compared with the Finnish growths charts [36], [37]. Height standard deviation (SD) score (height Z-score) was defined as deviation of height, in SD units, from the mean height for age and sex [37]. Weights were expressed as height-adjusted values, as percents of the mean in normal population of same sex and height, according to the Finnish standards [36], [37]. Based on questionnaire data and serum sex steroid concentrations pubertal development was scored either pre-, mid- or postpubertal by a pediatric endocrinologist (OM).

### Biochemistry

Blood samples and second void urine were collected at 8–10 am after an overnight fast between November and March (wintertime in Finland). Plasma calcium (Ca), phosphate (Pi) and alkaline phosphatase (ALP) were measured using standard methods. Reference ranges for plasma ALP were age-and sex-dependent and the measured values were transformed into Z-scores using normal values to allow for cross-sectional comparison. Serum 25-OHD was assayed with high-performance liquid chromatography (HPLC, evaluated Vitamin D External Quality Assessment Scheme, DEQAS), and plasma fasting parathyroid hormone (PTH) by an immunoluminometric method. Urinary concentrations of Ca, Pi and creatinine were analyzed using standard methods. All blood and urine measurements were analyzed in the Central Laboratory of Helsinki University Central Hospital.

### Bone and Body Composition Measurements

BMD, bone mineral content (BMC) and bone area (BA), were measured with dual-energy X-ray absorptiometry (DXA, Hologic Discovery A, pediatric software, version 12.4) from the lumbar spine (LS) (L1-L4), total hip and whole body (WB). DXA measurements were performed within three months of the biochemical sampling. All measured values were transformed into Z-scores using the equipment-specific age- and sex-adjusted reference data for US Caucasian children; all subjects were of normal height [38]. Body composition was analyzed with DXA to separate lean body mass and fat mass. Calibration of the measurements was performed by using a spine phantom; inter-CV% for the phantom BMC, area, and BMD were 0.35%, 0.21%, and 0.41%, respectively. The reproducibility of the DXA measurement for bone, fat, and lean mass is 1.2%, 1.9% and 0.7%, respectively, in children between 10 and 18 years of age [39]. Age and gender specific reference values [40] were utilized to derive Z-scores for fat percentage and lean mass.

### Statistical Analysis

Descriptive data are reported as medians and ranges, or as means ± SD. Association of variables was tested with Pearson or Spearman correlation. If a variable was not normally distributed, logarithmic transformation was applied. Partial correlation was used to illustrate the association after controlling for confounding factor(s). A one way analysis of variance, ANOVA was performed to comparisons between three or more groups, followed by Bonferroni (normally distributed data) or nonparametric Kruskall-Wallis (not normally distributed data). Analysis of covariance, ANCOVA was used to exclude the possibility that differences found with ANOVA are due to differences in relevant covariates. Before multiple linear regression analysis several variables were log-transformed to obtain (approximate) normal distribution (for instance PTH, calcium intake). Simple regression analysis was first performed to screen potential predictors for BMD and a multivariate stepwise linear regression model was used to identify and determine significant predictors for bone mass. Using the approach of stepwise variable selection, stepping up, only variables with a significance level of 0.05 were included in the model. All calculations were performed using SPSS version 15.0 for Windows. A p-value of less than 0.05 was considered statistically significant and p-values between 0.05 and 0.1 were considered to indicate trends.
